# In‐hospital outcomes in unhoused patients with cardiogenic shock in the United States: Insights from The National Inpatient Sample 2011−2019

**DOI:** 10.1002/clc.24235

**Published:** 2024-02-17

**Authors:** Ian Ergui, Joshua Salama, Urvashi Hooda, Bertrand Ebner, Michael Dangl, Louis Vincent, Rhea Sancassani, Rosario Colombo

**Affiliations:** ^1^ Division of Internal Medicine, Department of Medicine University of Miami Miller School of Medicine Miami Florida USA; ^2^ Division of Cardiology, Department of Medicine University of Miami Miller School of Medicine Miami Florida USA; ^3^ Department of Cardiology Jackson Memorial Hospital Miami Florida USA

**Keywords:** cardiogenic shock, homelessness, housing insecurity, mechanical circulatory support, unhoused

## Abstract

**Background:**

Unhoused patients face significant barriers to receiving health care in both the inpatient and outpatient settings. For unhoused patients with heart failure who are in extremis, there is a lack of data regarding in‐hospital outcomes and resource utilization in the setting of cardiogenic shock (CS).

**Hypothesis:**

Unhoused patients hospitalized with CS have increased mortality and decreased use of invasive therapies as compared to housed patients.

**Methods:**

The National Inpatient Sample (NIS) database was queried from 2011 to 2019 for relevant ICD‐9 and ICD‐10 codes to identify unhoused patients with an admission diagnosis of CS. Baseline characteristics and in‐hospital outcomes between patients were compared. Binary logistic regression was used to adjust outcomes for prespecified and significantly different baseline characteristics (*p* < .05).

**Results:**

We identified a weighted sample of 1 202 583 adult CS hospitalizations, of whom 4510 were unhoused (0.38%). There was no significant difference in the comorbidity adjusted odds of mortality between groups. Unhoused patients had lower odds of receiving mechanical circulatory support, left heart catheterization, percutaneous coronary intervention, or pulmonary artery catheterization. Unhoused patients had higher adjusted odds of infectious complications, undergoing intubation, or requiring restraints.

**Conclusions:**

These data suggest that, despite having fewer traditional comorbidities, unhoused patients have similar mortality and less access to more aggressive care than housed patients. Unhoused patients may experience under‐diuresis, or more conservative care strategies, as evidenced by the higher intubation rate in this population. Further studies are needed to elucidate long‐term outcomes and investigate systemic methods to ameliorate barriers to care in unhoused populations.

## Key points

- Unhoused patients hospitalized with cardiogenic shock (CS) have fewer traditional comorbidities however similar in‐hospital outcomes compared to housed patients.
- Unhoused patients with CS receive left and right heart catheterization and mechanical circulatory support less frequently than housed patients, however, undergo endotracheal intubation with greater frequency.
- A multidisciplinary approach along with strong community partnerships may result in improved access to care for this vulnerable population.

## Graphical abstract

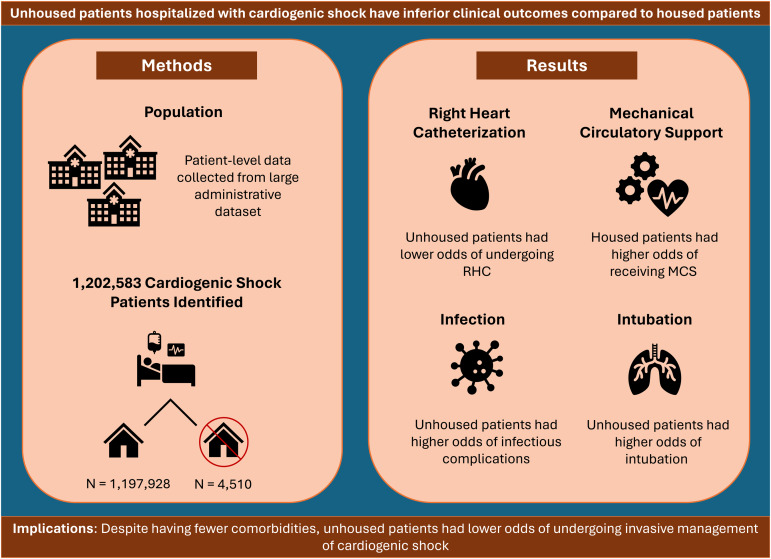

## 1 INTRODUCTION

In the United States (US), homelessness impacts up to 580 000 people on any given night.^1^ Unhoused patients suffer from decreased life expectancy,^2^ high morbidity and mortality, and significant barriers to receiving health care.^3^ Median housing costs have risen consistently for the past 10 years, and housing instability has been linked to poor cardiovascular health.^4^, ^5^ Governmental and institutional health care support for unhoused patients in the United States varies widely by region, and significant disparities in care exist.^6^ The average life expectancy of unhoused patients in the United States is 54.14 years, while patients in the top 1% of earnings have a life expectancy of up to 87.3 years.^7^ The leading cause of death in unhoused patients is cardiovascular disease.^8^ The terms unhoused, homeless, houseless and homelessness are used interchangeably.

A previous meta‐analysis showed that cardiovascular disease mortality was three times higher in unhoused patients in the inpatient setting.^9^ Unhoused patients admitted for acute myocardial infarction in major urban centers are less likely to undergo coronary angiography, percutaneous coronary intervention (PCI), or coronary artery bypass surgery.^3^ Hospitalized unhoused patients who survive an episode of cardiac arrest are less likely to undergo left heart catheterization or stent placement.^3^ Unhoused patients presenting with ST‐elevation myocardial infarction have a 3.83 times higher mortality than housed patients at PCI‐capable centers.^10^

Patients of low socioeconomic status (SES) with heart failure have higher hospitalization rates, higher rates of adverse cardiovascular events, and lower use of device therapies.^11^ One large retrospective study in a universal health care setting demonstrated that guideline‐directed medical therapy (GDMT) is utilized less frequently in low‐income patients.^12^ An analysis of the Atherosclerosis Risk in Communities database showed that, in the United States, low income and low education are associated with higher heart failure mortality and readmission rates.^13^

Patients admitted with cardiogenic shock (CS) represent the highest‐risk heart failure subgroup. Despite recent advances in interventional and medical care, short‐term inpatient mortality remains greater than 30% for patients with CS.^14^ Hospitalizations for CS have tripled between 2004 and 2018.^15^ The protocolized implementation of invasive hemodynamic monitoring and early mechanical circulatory support systems (MCS) may be associated with improved patient outcomes.^16^ However, the early recognition and aggressive use of MCS remains a significant challenge.^17^

Despite the increased burden of cardiovascular disease and decreased use of GDMT and invasive therapies in unhoused patients, there is a distinct lack of data regarding outcomes in unhoused patients with CS. We sought to investigate in‐hospital outcomes in unhoused patients with CS. We hypothesized that the unhoused population would have prolonged hospitalization, decreased MCS or invasive procedures, and higher odds of mortality, possibly due to a combination of financial and institutional barriers to optimal care.

## 2 MATERIALS AND METHODS

We conducted a retrospective study using the National Inpatient Sample (NIS) database. The NIS is maintained by the Health care Cost and Utilization Project (HCUP), a collaboration of federal, state, and industry partners sponsored by the Agency for Health care Research and Quality.

**Figure 1:**
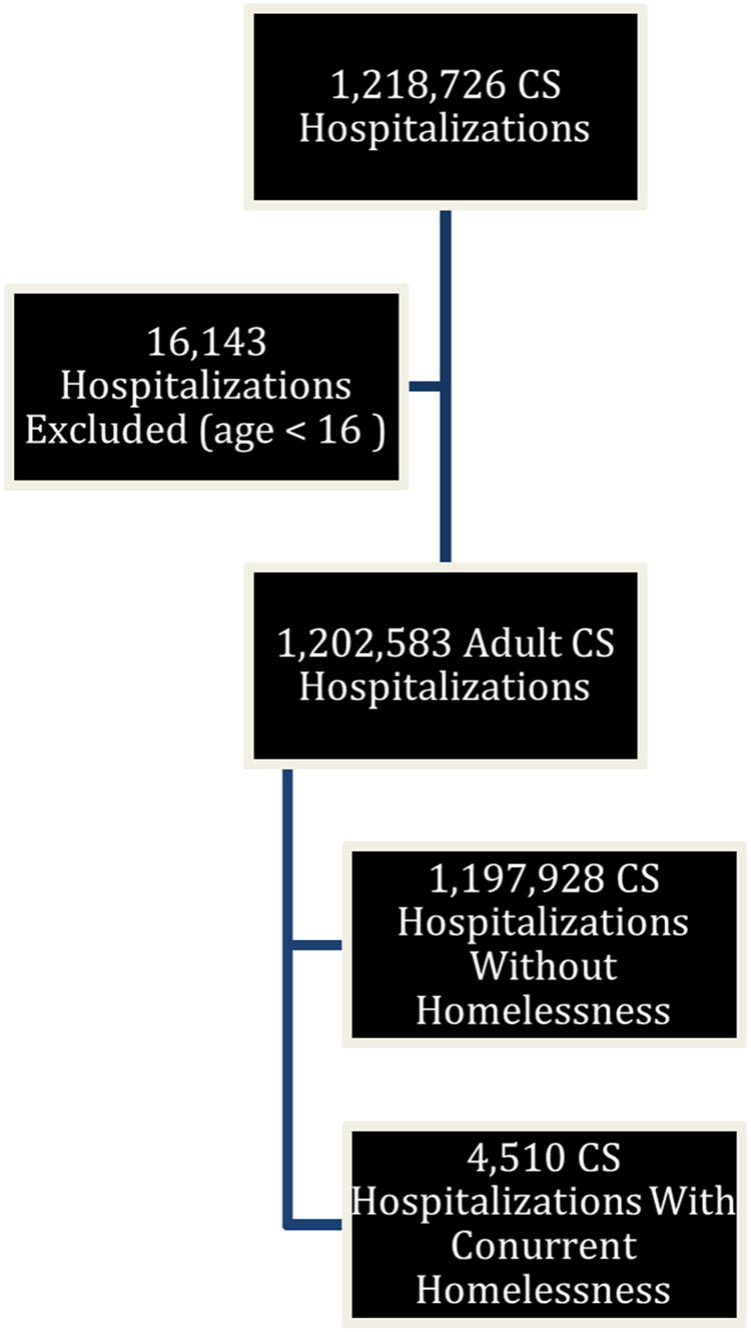
Flow chart detailing the selection of CS hospitalizations between 2011 and 2019 with and without homelessness. CS, cardiogenic shock.

The NIS was queried from January 1, 2011 to December 31, 2019, resulting in 324 130 692 weighted cases. Patients with a diagnosis of CS were identified based on relevant ICD‐9 or ICD‐10 codes (Supporting Information: Table 1). A total of 1 218 726 CS hospitalizations were identified, of which 4510 (0.4%) had concomitant homelessness (Supporting Information: Table 2). After excluding patients < 16 years of age (*N* = 16,143 patients), a total of 1 202 583 CS hospitalizations were included in the data analysis (Figure 1).

Baselines characteristics were evaluated including age, gender, race, elective admission status, and medical comorbidities (Table 1). The primary outcome was in‐hospital mortality. Secondary outcomes included the implementation of MCS, left heart catheterization, right heart catheterization, PCI, and the need for intubation. Additional secondary endpoints included cardiac arrest, stroke complications, infectious complications, vascular complications, length of stay, and cost of stay.

**Table 1:** Baseline characteristics of housed and unhoused patients admitted with cardiogenic shock.

Variable	Control Group (*n* = 1 197 928)	Unhoused (*n* = 4510)	*p* Value
Age	66.74 ± 14.46	53.82 ± 11.87	<.05
Female	38.3% (458 308)	20.4% (920)	<.05
Non‐White race	30.7% (368 074)	45.2% (2039)	<.05
Elective admission	10.6% (126 647)	2.8% (125)	<.05
Hypertension	74.8% (824 419)	61.2% (2505)	<.05
Diabetes mellitus	37.6% (447 804)	24.3% (1089)	<.05
Coronary artery disease	58.7% (698 404)	39.1% (1754)	<.05
Prior myocardial infarction	11.4% (136 461)	11.4% (514)	.74
Chronic kidney disease	35.9% (427 167)	21.9% (964)	<.05
On hemodialysis	3.9% (46 182)	1.7% (75)	<.05
History of heart failure	71.0% (850 221)	73.9% (3335)	<.05
Prior stroke	8.6% (103 369)	9.1% (410)	.27
Tobacco use	32.4% (388 097)	60.2% (2717)	<.05
Alcohol use	5.3% (62 494)	25.6% (1148)	<.05
Substance abuse	3.4% (40 950)	35.3% (1590)	<.05
Anemia	7.1% (63 172)	10.8% (409)	<.05
Asthma	4.4% (52 461)	6.0% (265)	<.05
Chronic obstructive pulmonary disease	23.3% (26 1350)	27.8% (1179)	<.05
Obese	14.9% (178 864)	8.0% (360)	<.05
Peripheral artery disease	9.6% (109 311)	5.0% (210)	<.05
Obstructive sleep apnea	9.6% (115 013)	5.1% (230)	<.05
Hyperlipidemia	45.2% (50 4052)	24.9% (1023)	<.05
Malignancy history	7.0% (84 122)	2.7% (120)	<.05
Chronic liver disease and cirrhosis	7.7% (89 833)	15.1% (665)	<.05

*Note*: Values are reported as mean ± standard deviation for continuous variables and percentage (number) for categorical variables. *p* Value ≤ .05 is considered significant (asterisk).

Descriptive statistics were used for continuous and categorical variables. Missing values for race were accounted for using multiple imputation as recommended by the HCUP. Mean and standard deviations are offered for parametric continuous variables. Median and interquartile range (IQR) are offered for nonparametric continuous variables. Percentages are given for categorical variables. Independent samples *t*‐tests were used for parametric continuous variables, and Pearson's chi‐square for categorical variables. The Mann−Whitney *U* test was used for nonparametric continuous variables. A *p* Value of <.05 was considered statistically significant. Multivariate logistic regression was utilized to develop adjusted odds ratios (aORs) and 95% confidence intervals to estimate the association between unhoused status and in‐hospital outcomes in CS hospitalizations. Models were adjusted for the prespecified covariates of sex, non‐White race, elective admission status, elderly patient status (defined as age ≥ 70 years), and medical comorbidities (Supporting Information: Table 3). Values were then recalculated under the assumptions of a probit model, namely normal distribution of sample errors. Adjusted probit regression was used to generate clinical outcome aORs and 95% confidence intervals as recommended by the HCUP.^18^ Outputs from the logistic and probit regressions were compared. Statistical analyses were performed using SPSS (IBM SPSS statistics for MAC, Version 26.0; IBM Corporation). Regression models were generated using generalized estimating equations.

## 3 RESULTS

Baselines characteristics of housed and unhoused patients are shown in Table 1. Housed patients were more likely to identify as women and were older than unhoused patients (Table 1). The housed group had a higher frequency of elective admissions. Unhoused patients were more likely to be non‐White. In terms of chronic conditions, housed patients were more likely to have hypertension, diabetes mellitus, coronary artery disease, chronic kidney disease, obesity, obstructive sleep apnea, hyperlipidemia, or a history of malignancy (Table 1). The housed group was additionally more likely to be on chronic hemodialysis.

Conversely, the unhoused group was more likely to have a history of tobacco use, alcohol abuse, or drug abuse (Table 1). Regarding chronic conditions, patients in the unhoused group had a higher frequency of heart failure, anemia, asthma, chronic obstructive pulmonary disease, and chronic liver disease (Table 1). There were no significant differences in the history of prior myocardial infarctions or strokes between groups.

Unadjusted in‐hospital events and outcomes are shown in Table 2. Patients with CS and homelessness had lower rates of mortality, cardiac arrest, stroke, bleeding complications, and vascular complications (Table 2). Unhoused patients had higher rates of infectious complications and deep venous thrombi/pulmonary emboli (Table 2).

**Table 2:** Events and outcomes for CS hospitalizations.

Variable	Control group (*n* = 1 197 928)	Unhoused (*n* = 4510)	*p* Value
Mortality	35.3% (422 651)	26.8% (1209)	<.05
Received mechanical circulatory support	20.9% (250 404)	9.1% (410)	<.05
Cardiac arrest	15.4% (184 200)	12.5% (564)	<.05
Stroke	5.1% (61 422)	3.7% (169)	<.05
Infectious complications	25.5% (305 650)	28.4% (1283)	<.05
All major bleeding	14.9% (178 221)	11.8% (530)	<.05
DVT/PE	6.7% (80 673)	8.1% (366)	<.05
Vascular complication	3.0% (35 621)	2.2% (100)	<.05
Left heart cath	31.3% (374 554)	21.3% (961)	<.05
Percutaneous coronary intervention	18.6% (222 344)	10.3% (465)	<.05
Pulmonary artery cath	9.6% (115 599)	8.0% (361)	<.05
Intubation	36.1% (432 694)	38.5% (1,737)	<.05
Palliative care consult	16.8% (201 128)	17.3% (781)	.34
Restraint use	3.2% (38 234)	7.1% (320)	<.05
Length of stay^a^	7.00 (IQR 3.0−14.0)	8.00 (IQR 4.0‐14.0)	<.05
Cost of stay^b^	$122 124.00 (IQR 57 469.0−249 082.0)	$119 225.00 (IQR 64 701.39−206 676.95)	<.05

*Note*: Outcomes reported as median (interquartile range) for nonparametric continuous variables and percentage (number) for categorical variables.

^a^ Length of stay reported in days.

^b^ Cost of stay reported in USD. *p* Value considered significant <.05 (asterisk).

Unhoused patients had a globally decreased frequency of interventions that were classified as procedural management of CS. Unhoused patients had lower rates of MCS, pulmonary artery catheterization, left heart catheterization, and PCI (Table 2). Notably, the rates of intubation were higher in the unhoused group (Table 2). Restraint use was also more frequent in the unhoused group (Table 2). There were no differences in the rates of palliative care consults between groups. The length of stay was longer in the unhoused group (median 8 days, IQR 4−14 vs. median 7 days, IQR 3−14, *p* < .05), as was the cost of stay (unhoused median $122 124.00 IQR 57 469.0− 249 082.0 vs. housed median 119 225.00 IQR 64 701.39−206 676.95, *p* < .05).

**Figure 2:**
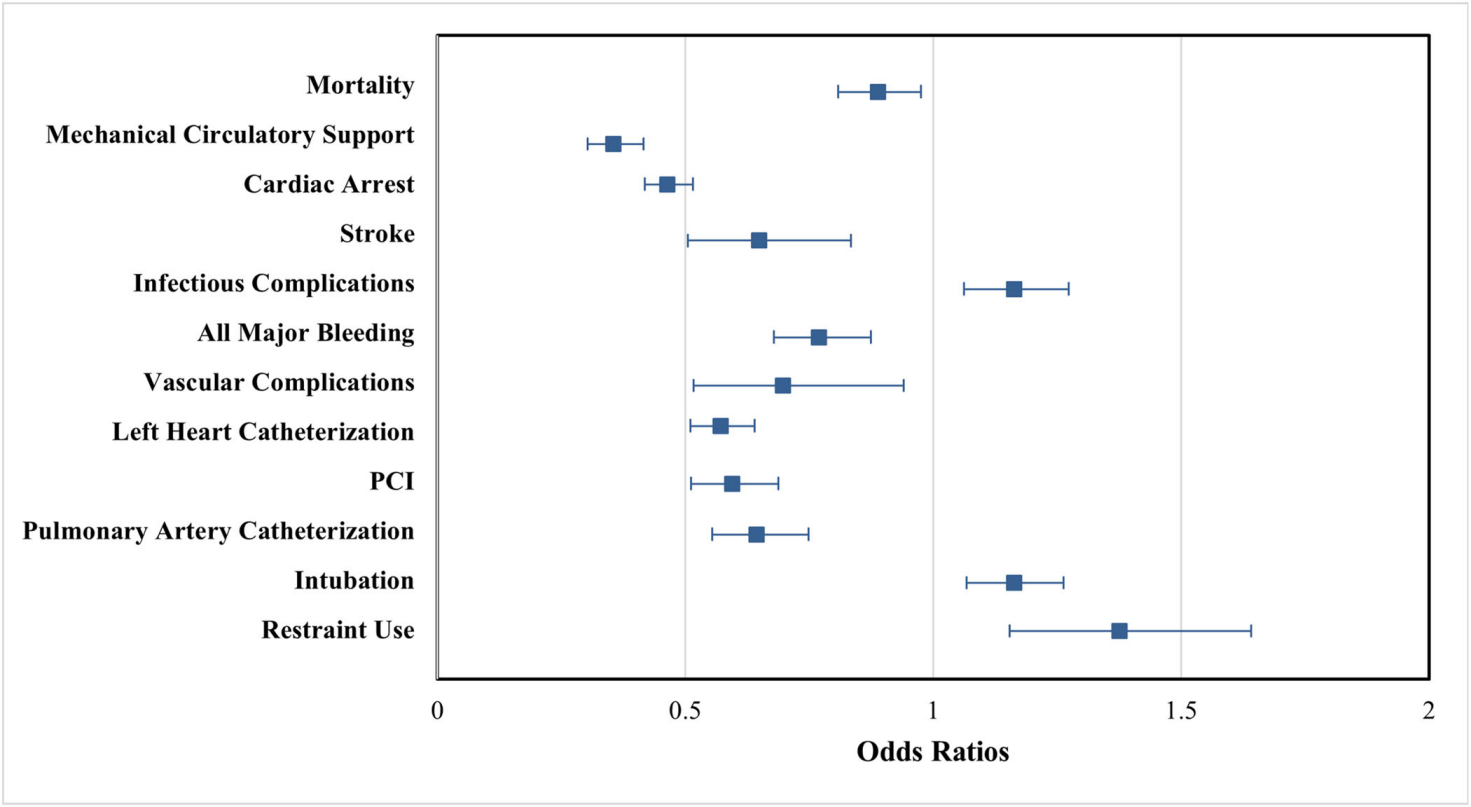
Adjusted in‐hospital outcomes and events in housed versus unhoused patients with CS. Values < 1 indicate increased odds in housed patients. CS, cardiogenic shock.

A binary logistic regression model was used to adjust for the prespecified covariates and comorbidities (Figure 2). After adjustment, there were lower odds of death (aOR 0.89, 95% CI 0.81−0.98, *p* < .05) and vascular complications (aOR 0.69, 95% CI 0.52−0.94, *p* < .05) in the unhoused group. Unhoused patients had lower odds of cardiac arrest (aOR 0.46, 95% CI 0.42−0.52, *p* < .05), stroke (aOR 0.65, 95% CI 0.51−0.83, *p* < .05), and bleeding complications (aOR 0.77, 95% CI 0.68−0.87, *p* < .05). The unhoused cohort had significantly higher odds of infectious complications (aOR 1.16, 95% CI 1.06−1.27, *p* < .05). Unhoused patients were less likely to receive MCS (aOR 0.36, 95% CI 0.30−0.42, *p* < .05), left heart catheterization (aOR 0.57, 95% CI 0.51−0.64, *p* < .05), PCI (aOR 0.59, 95% CI 0.51−0.69, *p* < .05), or pulmonary artery catheterization (aOR 0.64, 95% CI 0.55−0.75, *p* < .05). Interestingly, the unhoused cohort had significantly higher odds of undergoing intubation (aOR 1.16, 95% CI 1.07−1.26, *p* < .05) or requiring restraints (aOR 1.38, 95% CI 1.15−1.64, *p* < .05).

Clinical outcomes were re‐evaluated using a probit regression model that was adjusted for the prespecified covariates and comorbidities (Figure 2). Outputs from the probit model were compared to those from the binary logistic model to evaluate differences in outcome measures when assuming a normal versus logistic distribution of errors in the sample. In the probit model, unhoused patients had slightly lower odds of death (aOR 0.94, 95% CI 0.89−0.99, *p* < .05). Unhoused patients had lower odds of vascular complications and cardiac arrests, however unlike in the binary logistic model these values did not meet the threshold for statistical significance (vascular complications aOR 0.893, 95% CI 0.791−1.008, *p* = .07: cardiac arrest aOR 0.94, 95% CI 0.88−1.01, *p* = .08). Unhoused patients had lower odds of stroke (aOR 0.77, 95% CI 0.69−0.87, *p* < .05) and bleeding complications (aOR 0.82, 95% CI 0.77−0.88, *p* < .05) in the probit model, consistent with findings from the logistic regression.

Unhoused patients had lower odds of undergoing pulmonary artery catheterization (aOR 0.78, 95% CI 0.71−0.86, *p* < .05), left heart catheterization (aOR 0.72, 95% CI 0.67−0.77, *p* < .05), PCI (aOR 0.33, 95% CI 0.32−0.22, *p* < .05) and MCS (aOR 0.59, 95% CI 0.54−0.64, *p* < .05) compared to housed patients in the probit model. Unhoused patients had higher odds of intubation (aOR 1.08, 95% CI 1.03−1.14, *p* < .05) and restraint use (aOR 1.16, 95% CI 1.07−1.23, *p* < .05) in the probit model as well. These values were consistent with the findings in the binary logistic regression model.

## 4 DISCUSSION

We present the only nationwide retrospective analysis of CS outcomes among patients with housing insecurity to date. Prior studies have shown that low SES is associated with lower use of MCS devices in patients with CS.^19^, ^20^ Unhoused adults admitted for cardiovascular conditions have historically suffered from higher inpatient morbidity and mortality than the housed.^3^ Comorbidities thought to contribute to the burden of cardiovascular disease in unhoused patients include cigarette smoking, poor control of hypertension and diabetes, alcohol abuse, substance abuse, and medication nonadherence.^21^ Advances in medical and device therapies over the past 30 years have revolutionized the care of heart failure patients,^22^ however evidence suggests that unhoused patients of low SES are not seeing these benefits.^23^

We demonstrate that, overall, invasive hemodynamic monitoring via pulmonary artery cannulation and MCS occurred less frequently in unhoused patients. Racial minorities have been shown to receive MCS less frequently than White patients, and the higher frequency of non‐White patients in the unhoused group may be an effect modifier for the observations in this study.^24^, ^25^ Despite unhoused patients being younger and having fewer comorbidities than their housed counterparts, comorbidity‐adjusted odds of mortality remained comparable between both groups across various regression models. Unhoused patients may have delayed time to presentation when in the acute phase of heart failure and may present with a sicker phenotype than housed patients. Endotracheal intubation, interestingly, occurred more frequently in the unhoused patient group despite the younger age and decreased presence of medical comorbidities such as hypertension, diabetes mellitus, coronary artery disease, sleep apnea, and obesity in this group. This observation may be explained by increased pulmonary edema from underdiuresis, increased smoking/drug abuse, behavioral dyscrasias, an overall more conservative approach to management in this population, or a combination of the aforementioned factors. Positive pressure ventilation can decrease preload and increase afterload in patients with right ventricular failure, and efforts should be made to prevent patient deterioration to the point of intubation where possible.^26^

Patients with CS can present with a range of symptoms, from subtle signs of hypoperfusion to hemodynamic instability necessitating MCS. The optimal treatment of CS patients remains a subject of debate, however early revascularization for MI‐driven CS, vasopressor/inotrope therapy, and temporary MCS with or without hemodynamic monitoring in select patients are tools in the armamentarium of clinicians tasked with treating this deadly condition.^27^

Unhoused patients, when interviewed, endorse that stigmatization from health care professionals, tradeoffs between basic needs and medications, and situational instability are some of the unique challenges faced.^28^ Concerns regarding long‐term medication adherence limit the use of coronary revascularization in low SES and unhoused patients in the community.^29^, ^30^ Collaboration between health care and social service sectors are needed to see meaningful improvements in care for the unhoused.^31^ Access to affordable housing has been shown to improve medication adherence in unhoused populations.^32^ Social interventions such as income assistance, harm reduction, and mental health support improve morbidity and mortality in unhoused populations.^33^

The current study has several limitations. The low sample size of unhoused CS patients ensures that any observations drawn from this study are mainly descriptive and causation cannot be inferred. The granularity of ICD‐9 and ICD‐10 codes makes assessing the severity and etiology of CS in an individual patient record impossible. Patients with ischemia driven CS have increased mortality and worse in‐hospital outcomes compared to patients with heart failure driven shock.^34^ We attempted to adjust for this potential confounder by including covariates for heart failure, coronary artery disease and peripheral artery disease (a coronary artery disease equivalent) in our binary logistic and probit regression models. Patient level descriptors such as blood pressure, heart rate, perfusion status, urine output, and biochemical markers such as pro‐BNP and troponin are not available for review. We are unable to ascertain the cardiac output, mean arterial pressure, cardiac index, or cardiac power index of the patients in this study. Information regarding echocardiographic readings or predictors of poor outcome are not offered.

The disparity between the overall increased incidence of traditional cardiac comorbidities seen in the housed population in this study, however the decreased implementation of aggressive CS therapy and increased frequency of intubation in the unhoused population is a clear gap that health care institutions must bridge to ensure equal care for the most vulnerable patients. Heart failure teams should consider a multidisciplinary approach to evaluating unhoused patients with CS.

## 5 CONCLUSION

Unhoused patients with CS have comparable comorbidity‐adjusted mortality to housed populations despite being an overall younger and healthier cohort. When adjusted for comorbidities, homelessness was independently associated with a decreased likelihood of undergoing invasive procedures and increased likelihood of endotracheal intubation. Further studies are needed to develop health care delivery models to address these disparities in care.

## CONFLICT OF INTEREST STATEMENT

The author declare no conflict of interest.

## Supporting information

Supporting information.

## Data Availability

The data that support the findings of this study are available in National Inpatient Sample at https://hcup-us.ahrq.gov/nisoverview.jsp. These data were derived from the following resources available in the public domain: ‐ Health care Cost and Utilization Project, https://hcup-us.ahrq.gov/tech_assist/centdist.jsp.
