# Supplementary material for: In‐hospital outcomes in unhoused patients with cardiogenic shock in the United States: Insights from The National Inpatient Sample 2011−2019
Source: Clin Cardiol. 2024 Feb 17;47(2):e24235. doi: 10.1002/clc.24235 (PMC10873680; doi:10.1002/clc.24235)
Supplement: Supplementary file 1 — Supporting information. [file CLC-47-e24235-s001.docx]

**Supplemental Material:**

The NIS contains data on inpatient hospitalizations from participating states (n=47 and the District of Columbia). This is the largest all-payers inpatient dataset, approximating a 20% sample of US hospitalizations from community hospitals (defined as general or subspecialty short term hospitals, excluding rehabilitation and long-term acute care facilities). Over 1000 hospitals contribute data from about 7 million discharges annually. The NIS approximates more than 97% of the US population. Patient, hospital, and state level identifiers are excluded from the database. Institutional review board approval was unnecessary as all information in the NIS is deidentified. Discharge weights are provided with all records to derive national estimates. All data were weighted as recommended by the NIS to derive national estimates. Prior to October 1, 2015, hospital administrative data in the US was encoded using the International Classification of Diseases, Ninth Edition, Clinical Modification/Procedure Coding System (ICD-10-CM/PCS). The last 3 months of 2015, and from 2016 onwards, the NIS contains data based on ICD-10-CM/PCS codes. These changes are accounted for in the substructure of the NIS.

Binary logistic and probit regression models in this study were adjusted for hypertension, diabetes mellitus, history of coronary artery disease, history of myocardial infarction, chronic kidney disease, hemodialysis, history of heart failure, tobacco use, alcohol use, substance abuse, anemia, asthma, COPD, obesity, stroke history, chronic liver disease/ cirrhosis, end stage renal disease, hyperlipidemia, malignancy history, obstructive sleep apnea, peripheral artery disease.

| **Variable** | **ICD 9 and 10 Codes** |
| --- | --- |
| Cardiogenic shock | R570, 78551 |

**Supplemental Table 1:** Codes used to identify cardiogenic shock hospitalizations were exclusively applied to the diagnostic section of the NIS database to select for the cardiogenic shock admissions.

| **Variables** | **ICD 9 and 10 Codes** |
| --- | --- |
| Homelessness | Z590 |
| Homelessness unspecified | Z5900 |
| Sheltered homelessness | Z5901 |
| Unsheltered homelessness | Z5902 |
| Inadequate housing | Z591 |
| Housing instability, housed, with risk of homelessness | Z59811 |
| Housing instability, housed, homelessness in past 12 months | Z59812 |
| LACK OF HOUSING | V600 |
| INADEQUATE HOUSING | V601 |

**Supplemental Table 2:** Diagnostic codes used to identify unhoused patients.

| **Variables** | **ICD-9 and ICD-10 codes** |
| --- | --- |
| Cardiogenic Shock | 78551, R570 |
| Hypertension | 401.X, 402.X, 405.X, I10, I11.X, I12.X, I15.X |
| Diabetes Mellitus | 250.X, E08.X, E09.X, E10.X, E11.X, E12.X, E13.X, E14.X |
| Coronary Artery Disease | 412.X, 414.0X, 414.2, 414.3, 414.4, 414.8, 414.9, I25.1X, I25.2, I25.5, I25.6, I25.8X, I25.9 |
| History of Myocardial Infarctions | 412, I25.2 |
| Chronic Kidney Disease | 585.X, N18.X |
| Renal Replacement Therapy with Hemodialysis | 39.95. 5A1D00Z, 5A1D60Z, 5A1D70Z 5A1D80Z, 5A1D90Z |
| History of Heart Failure | 40201, 40211, 40291, 40401, 40403, 40411, 40413, 40491, 40493, I110, I130, I132, 39891, 4254, 4255, 4256, 4257, 4258, 4259, I099, I255, I420, I425, I426, I427, I428, I429, P290, I43 |
| Stroke History | 438.X, I69.X, V12.54, Z86.73 |
| Tobacco | 305.1, 989.84, V15.82, F17.X, T65.2X, Z72.0, Z87.891 |
| Alcohol | 291.1, 291.2, 291.3, 291.5, 291.6, 291.7, 291.81, 291.89, 291.9, 265.2, 303.00, 303.90, 303.91, 303.92, 303.93, 305.00, 305.01, 305.02, 305.03, 357.5, 425.5, 535.3, 571.0, 571.1, 571.2, 571.3, 980.X, E52, F10.X, G62.1, I42.6, K29.2, K70.0, K70.3, K70.9, T51.X, V11.3, Z50.2, Z71.4, Z72.1, |
| Substance abuse | 292.X, 304.X, 305.2, 305.3, 305.4, 305.5, 305.6, 305.7, 305.8, 305.9, F11.X, F12.X, F13.X, F14.X, F15.X, F16.X, F18.X, F19.X, V65.42, Z71.5, Z72.2 |
| Anemia | 280.X, 281.X, D50.X, D51.X, D52.X, D53.X |
| Asthma | 493.X, J45.X |
| Chronic Obstructive Pulmonary Disease | 490, 491.X, 492.X, 496, J40, J41.X, J42, J43.X, J44.X |
| Obesity | 278.00, 278.01, 278.03, E66.01, E66.09, E66.1, E66.2, E66.8, E66.9 |
| Peripheral Artery disease | 443.9, 440.X, I70.X, I73.9 |
| Obstructive Sleep Apnea | 78057, 32720, 32723, 32729, 78051, 78603, 78053, 32700, 32726, G4730, G4733, G4739, R0681 |
| Dyslipidemia | 272.X, E78.X |
| Malignancy History | Z85.X, V10.X |
| Chronic Liver Disease and Cirrhosis | 571.X, 573.X, 572.2, 572.3, 572.4, 572.8, K70.2, K70.9, K703.X, K71.3, K71.4, K71.5X, K71.7, K71.8, K71.9, K71.6, K72.1X, K72.9X, K73.X, K74.X, K74.6X, K75.3, K75.4, K75.9, K75.81, K75.89, K76.X |
| Received Mechanical Circulatory support | 5A15223, 3965, 3760, 3762, 3765, 3768, 02HA0RS, 02HA3RS, 02HA4RS, 5A02116, 5A02216, 02HA0RZ, 02HA3RZ, 02HA4RZ, 02HA0RJ, 02HA3RJ, 02HA4RJ, 5A0211D, 5A0221D, 5A02210, 5A02110, 3761, 5A02115 |
| Cardiac Arrest | 4275, I46.X |
| Stroke Complications | 430, I60.X, 431, 432.9, I61.X, 432.X, I62.X, 433.X, 434.X, 435.X, 436, 432.9, I63.X |
| Infectious Complications | 038.X, 995.91, 995.92, A40.X, A41.X, R65.2, 785.52, 998.02, T81.12, 998.51, 998.59, T814XXA, 996.60, 996.61, T826XXA |
| All Major Bleeding | 998.11, 998.12, I97.621, I97.630, I97.631, I97.638, I97.410, I97.411, I97.418, I97.42, I97.410, I97.411, I97.418, I97.42, I97.610, I97.611, I97.618, I97.620, 568.81, K66.1, 456.0, 456.20, 530.21, 530.7, 530.82, 531.00, 531.01, 531.20, 531.21, 531.40, 531.41, 531.60, 531.61, 532.00, 532.01, 532.20, 532.31, 532.40, 532.41, 532.60, 532.61, 533.00, 533.01, 533.20, 533.21, 533.40, 533.41, 533.60, 533.61, 534.00, 534.01, 534.20, 534.21, 534.40, 534.41, 534.60, 534.61, 535.01, 535.11, 535.21, 535.31, 535.41, 535.51, 535.61, 535.71, 537.83, 537.84, 562.02, 562.03, 562.12, 562.13, 569.3, 569.85, 569.86, 578.X, I85.01, I85.11, K22.11, K22.6, K22.8, K25.0, K25.2, K25.4, K25.6, K26.0, K26.2, K26.4, K26.6, K27.0, K27.2, K27.4, K27.6, K28.0, K28.2, K28.4, K28.6, K29.01, K29.21, K29.31, K29.41, K29.51, K29.61, K29.71, K29.81, K29.91, K31.811, K57.11, K57.13, K57.31, K57.33, K62.5, K55.21, K31.82, K63.81, K92.0, K92.1, K92.2, 596.7, 599.70, 599.71, N32.89, R31.9, R31.0, 786.30, 786.39, R04.2, R04.9, 784.7, R04.0, 459.0, R58 |
| DVT/PE | 415.11, 415.13, 415.19, 451.81, 451.9, 453.40, 453.41, 453.42, 453.8, 453.9, I82.491, I82.492, I82.493, I82.499, I82.409, I82.621, I82.622, I82.629, I82.401, I82.402, I82.623, I82.419, I824Y1, I82.4Y2, I82.4Y3, I82.403, I82.4Z1, I82.4Z2, I82.4Z3, I82.429, I82.A19, I82.890, I82.423, I82.439, I82.449, I82.413, I82.411, I82.412, I82.A11, I82.A12, I82.C11, I82.C12, I82.433, I82.443, I82.A13, I82.432, I82.441, I82.442, I82.C19, I82.421, I82.422, I82.C13, I82.431, I82.B19, I82.B13, I82.B11, I82.B12, I82.611, I82.612, I82.220, I82619, I82.613, I82.90, I82.4Y9, I82.4Z9, I82.649, I82.3, I81, I82.210, T8172XA, I82601, I82.602, I82.603, I97.89, I82.609, I82.451, I82.451, I82.452, I82.453, I82.459, I82.461, I82.462, I82.290, I26.99, I26.93, I26.02, I26.09 |
| Vascular Complications | 9982, 9992, 99771, 99772, 99779, 4470, I9751, I9752, T801XXA, T81710A, T81711A, T81718A, T8172XA, I770, 3956, 3931, 3941, 3949, 3952, 3957, 3959, 3979, 02QP0ZZ, 02QP3ZZ, 02QP4ZZ, 02QQ0ZZ, 02QQ3ZZ, 02QQ4ZZ, 02QR0ZZ, 02QR3ZZ, 02QR4ZZ, 02QW0ZZ, 02QW3ZZ, 02QW4ZZ, 02QX0ZZ, 02QX3ZZ, 02QX4ZZ, 02QS0ZZ, 02QS3ZZ, 02QS4ZZ, 02QV0ZZ, 02QV3ZZ, 02QV4ZZ, 900.X, 901.X, 902.X, 903.X, 904.X, 03Q.X, 04Q.X, 0W3.X, 02UP.X, 02UQ.X, 02U3.X, 02UR.X, 02US.X, 02UT,X, 02UV.X, 02UW.X, 03U.X, 03U.X, |
| Left Heart Catheterization | B210010, B2100ZZ, B210110, B2101ZZ, B210Y10, B210YZZ, B211010, B2110ZZ,B211110, B2111ZZ, B211Y10, B211YZZ, B212010,B2120ZZ, B212110, B2121ZZ, B212Y10, B212YZZ,B213010, B2130ZZ, B213110,B2131ZZ, B213Y10, B213YZZ, B2150ZZ, B2151ZZ,B215YZZ, B2160ZZ, B2161ZZ,B216YZZ, B2170ZZ, B2171ZZ, B217YZZ, B2180ZZ,B2181ZZ, B218YZZ, B21F0ZZ,B21F1ZZ, B21FYZZ, 8850, 8853, 8854, 8855, 8856, 8857, 8859, 8858 |
| Percutaneous Coronary Intervention | 3606, 3607, 3609, 0066, 1755, 02703ZZ, 02704ZZ, 02713ZZ, 02714ZZ, 02723ZZ, 02724ZZ, 02733ZZ, 02734ZZ, 02Q03ZZ, 02Q04ZZ, 02Q13ZZ, 02Q14ZZ, 02Q23ZZ,  02Q24ZZ, 02Q33ZZ, 02Q34ZZ, 0270346, 027034Z, 0270356, 027035Z, 0270366,  027036Z, 0270376, 027037Z, 02703D6, 02703DZ, 02703E6, 02703EZ, 02703F6,  02703FZ, 02703G6, 02703GZ, 02703T6, 02703TZ, 02703Z6, 0270446, 027044Z,  0270456, 027045Z, 0270466, 027046Z, 0270476, 027047Z, 02704D6, 02704DZ,  02704E6, 02704EZ, 02704F6, 02704FZ, 02704G6, 02704GZ, 02704T6, 02704TZ,  02704Z6, 0271346, 027134Z, 0271356, 027135Z, 0271366, 027136Z, 0271376,  027137Z, 02713D6, 02713DZ, 02713E6, 02713EZ, 02713F6, 02713FZ, 02713G6,  02713GZ, 02713T6, 02713TZ, 02713Z6, 0271446, 027144Z, 0271456, 027145Z,  0271466, 027146Z, 0271476, 027147Z, 02714D6, 02714DZ, 02714E6, 02714EZ,  02714F6, 02714FZ, 02714G6, 02714GZ, 02714T6, 02714TZ, 02714Z6, 02714ZZ,  0272346, 027234Z, 0272356, 027235Z, 0272366, 027236Z, 0272376, 027237Z,  02723D6, 02723DZ, 02723E6, 02723EZ, 02723F6, 02723FZ, 02723G6, 02723GZ,  02723T6, 02723TZ, 02723Z6, 0272446, 027244Z, 0272456, 027245Z, 0272466,  027246Z, 0272476, 027247Z, 02724D6, 02724DZ, 02724E6, 02724EZ, 02724F6,  02724FZ, 02724G6, 02724GZ, 02724T6, 02724TZ, 02724Z6, 0273346, 027334Z,  0273356, 027335Z, 0273366, 027336Z, 0273376, 027337Z, 02733D6, 02733DZ,  02733E6, 02733EZ, 02733F6, 02733FZ, 02733G6, 02733GZ, 02733T6, 02733TZ,  02733Z6, 02733ZZ, 0273446, 027344Z, 0273456, 027345Z, 0273466, 027346Z,  0273476, 027347Z, 02734D6, 02734DZ, 02734E6, 02734EZ, 02734F6, 02734FZ,  02734G6, 02734GZ, 02734T6, 02734TZ, 02734Z6, 02734ZZ, 02Q03ZZ, 02Q04ZZ, 02Q13ZZ, 02Q14ZZ, 02Q23ZZ, 02Q24ZZ, 02Q33ZZ, 02Q34ZZ, |
| Pulmonary Artery Catheterization | 02HQ32Z, 02HQ42Z, 02HR00Z, 02HQ30Z, 02HP40Z, 02HP32Z, 02HP30Z, 02HQ0YZ, 02HP3DZ, 02HQ3YZ, 02HQ40Z, 02HQ42Z, 02HQ43Z, 02HQ4DZ, 02HQ4YZ, 02HR30Z, 02HR32Z, 02HR3DZ, 02HR3YZ, 02HR40Z, 02HR42Z, 02HR4DZ, 02HR4YZ, 4A023N6, 3721 |
| Intubation | 0BH17EZ, 0BH13EZ, 0BH18EZ, 9604, 9605 |
| Palliative Care Consult | Z515, V667 |
| Restraint Use | Z781, V4987 |

**Supplemental Table 3:** ICD-9-CM/PCS and ICD-10-CM/PCS codes for comorbidities and CS complications.
